# Effects of maternal fructose intake on the offspring’s kidneys

**DOI:** 10.3389/fphys.2022.969048

**Published:** 2022-09-06

**Authors:** Rogério Argeri, Erika Emy Nishi, Débora Conte Kimura Lichtenecker, Guiomar Nascimento Gomes

**Affiliations:** ^1^ Department of Physiology, Escola Paulista de Medicina, Federal University of São Paulo, São Paulo, Brazil; ^2^ Postgraduate Program in Translational Medicine, Department of Medicine, Escola Paulista de Medicina, Federal University of São Paulo, São Paulo, Brazil

**Keywords:** maternal fructose consumption, renin, angiotensin, aldosterone system, renal function, effect on the offspring, glomerular enlargement

## Abstract

Fructose overload is associated with cardiovascular and metabolic disorders. During pregnancy, these alterations may affect the maternal environment and predispose offspring to diseases. Aims: To evaluate the renal morphology and function of offspring of dams that received fructose overload during pregnancy and lactation. Methods: Female Wistar rats were divided into the control (C) and fructose (F) groups. C received food and water *ad libitum*, and F received food and d-fructose solution (20%) *ad libitum*. The d-fructose offer started 1 week before mating and continued during pregnancy and lactation. The progeny were designated as control (C) or fructose (F); after weaning, half of the F received water to drink (FW), and half received d-fructose (FF). Blood pressure (BP) and renal function were evaluated. The expression of sodium transporters (NHE3-exchanger, NKCC2 and NCC-cotransporters, and ENaC channels) and markers of renal dysfunction, including ED1 (macrophage), eNOS, 8OHdG (oxidative stress), renin, and ACE 1 and 2, were evaluated. CEUA-UNIFESP: 2757270117. The FF group presented with reduced glomerular filtration rate and urinary osmolarity, increased BP, proteinuria, glomerular hypertrophy, macrophage infiltration, and increased expression of transporters (NHE3, NCC, and ENaC), 8OHdG, renin, and ACE1. The FW group did not show increased BP and renal functional alterations; however, it presented glomerular hypertrophy, macrophage infiltration, and increased expression of the transporters (NHE3, NKCC2, NCC, and ENaC), renin, and ACE1. These data suggest that fructose overload during fetal development alters renal development, resulting in the increased expression of renin, ACE1, and sodium transporters, thus predisposing to hypertension and renal dysfunction.

## Introduction

Hypertension is a significant public health problem owing to its high prevalence and association with cardiovascular and renal diseases (Mills et al., 2016). This multifactorial disease may manifest as a result of insults during critical periods of fetal development (Franco et al., 2003; Rocha et al., 2005; Zandi-Nejad et al., 2006; Magaton et al., 2007; Thomal et al., 2010; Jones et al., 2012; Argeri et al., 2016). Studies have shown that during pregnancy, nutritional restriction (Franco et al., 2003; Zandi-Nejad et al., 2006; Jones et al., 2012), hyperglycemia (Rocha et al., 2005; Magaton et al., 2007), and sleep restriction (Thomal et al., 2010; Argeri et al., 2016) alter fetal growth predisposing to the development of hypertension in adulthood. Thus, identifying other conditions that can disturb the intrauterine environment is essential to prevent the development of arterial hypertension through fetal programming.

Fructose is widely used in processed foods such as soft drinks, jellies, cakes, and puddings (Bray et al., 2004; Bray, 2008). Increased consumption of this sugar has been associated with arterial hypertension and metabolic dysfunction (glucose intolerance and increased plasma insulin levels, among others) (Navarro-Cid et al., 1995; Farah et al., 2006; Cunha et al., 2007; Brito et al., 2008; De Angelis et al., 2012). These alterations caused by the high consumption of fructose during pregnancy can predispose offspring to non-communicable diseases. Significant alterations in glucose metabolism were observed in the offspring of rats that consumed large amounts of fructose during pregnancy and lactation, thus confirming this hypothesis (Sloboda et al., 2014; Song et al., 2017). Regarding the effects on the kidneys, changes in the expression of genes involved in renal morphogenesis were observed in the offspring of rats subjected to high fructose consumption (Tain et al., 2015b), and altered expression of enzymes related to oxidative stress (iNOS and COX-2) was observed in the renal tissue (Sarı et al., 2015).

Several experimental models have evaluated the effects of maternal high fructose consumption, alone or in combination with a high sodium and/or high fat diet, or with different doses of fructose (usually very high doses) (Olatunji et al., 2001; Abdulla et al., 2011; Tain et al., 2015a; Rodrigo et al., 2016; Saad et al., 2016; Fauste et al., 2021); however, few have evaluated in detail the effects on the structural and functional alterations of the kidneys from the offspring of rats subjected only to fructose overload.

The present study aimed to evaluate renal function and morphology and the expression of sodium transporters and markers of renal dysfunction in the kidneys of offspring of dams that received fructose overload during pregnancy and lactation. In addition, half of the offspring of dams that received fructose until lactation continued to receive fructose until adulthood.

This experimental group presented a significant increase in blood pressure, important renal morphology, and functional changes. The group of offspring exposed to fructose only in the pre-and perinatal periods did not show many functional changes, probably due to adaptive processes; however, the morphological changes found in this group demonstrated that exposure to fructose during nephron formation could be a threat to renal integrity, which may lead to future consequences.

## Methods

The study was approved by the Ethical Research Committee of the Universidade Federal de Sao Paulo (protocol 7647020614) and adhered to international guidelines for the care of animals. Twelve-week-old female Wistar rats were obtained from the Centro de Desenvolvimento de Modelos Experimentais Para Biologia e Medicina (CEDEME) University Animal Breeding Center. The rats were kept in a temperature-controlled room (22°C) with lights on from 7 a.m. to 7 p.m.

### Summary of the experimental protocol:

1) Female rats were distributed into Control and Fructose groups;
2) Male and Female rats were caged together for mating;
3) Pregnancy was confirmed by the presence of sperm in vaginal smears;
4) Weaning occurred 21 days after birth;
5) Renal function and blood pressure were evaluated in the offspring at 4 months of age.

### Experimental design

Female rats were randomly assigned to control or fructose groups: C, the control group and F, fructose group (subjected to fructose overload during pregnancy and lactation). Rats in the control group received food and water *ad libitum*. The fructose group received food and drinking solution containing 20% d-fructose *ad libitum* (d-Fructose, Labsynth, Diadema-S.P. Brazil). The amount of fructose offered to dams using different protocols may vary widely (10%–60%) (Abdulla et al., 2011). The dose used in the present study was 20% because it is relatively low, although it has been shown to affect blood pressure (Bernardes et al., 2018). One week before mating, fructose solution was offered to the fructose group and continued to be available during pregnancy and lactation for 24 h/day until weaning. Rats in groups C and F were divided into pairs and caged overnight with a male to mate, and vaginal smears were collected the following morning. The presence of sperm was considered a positive result. After birth, litter size was standardized to eight pups per litter. The offspring remained with the dams for 21 days. After weaning, pups were separated and placed in collective cages.

The offspring formed three experimental groups:

C: Male offspring from control dams (eight animals) that received water to drink.

FW: Male offspring from fructose dams (subjected to fructose overload during pregnancy and lactation) (eight animals) that received water to drink after weaning.

FF: Male offspring from fructose dams (subjected to fructose overload during pregnancy and lactation) (eight animals) continued to receive fructose solution to drink after weaning.

All groups received food *ad libitum*.

At 4 months of age, the animals were weighed, systolic blood pressure (BP) was measured, and renal function was evaluated.

### Measurement of systolic blood pressure

BP was measured using the indirect technique of tail plethysmography. The rat was placed in an acrylic containment cylinder with only the tail remaining exposed. Then, the containment cylinder was placed in a heating chamber at a constant temperature of 34°C to promote slight dilatation of the caudal artery. The sphygmomanometer, with a sensor connected to a recording system, was coupled to the proximal portion of the rat tail (caudal artery) (tail plethysmography, IITC Life Science Inc. Woodland Hills, CA, United States). The cuff was inflated to 220 mmHg and slowly deflated, and the systolic pressure was recorded. For each rat, at least three measurements were taken in a row, with the average being considered the blood pressure value.

### Evaluation of renal function—clearance evaluation

Before the clearance evaluation, the rats were placed in metabolic cages (Criffa, Barcelona, Spain) for 24 h. Urine samples were collected to measure protein excretion and urinary osmolality (Uosm).

For clearance evaluation, the rats were anesthetized with sodium thiopental (Cristália) at a dose of 60 mg/kg i.p. and additional doses were administered during the experiment whenever the anesthetic plane of the animals became superficial.

Initially, a polyethylene tube (PE 260) was inserted into the trachea of each animal to maintain ventilation. The right carotid artery was catheterized with PE 20 polyethylene for blood sampling, and the left external jugular artery was catheterized with PE 50 polyethylene for infusion of different solutions. A catheter (PE 260 polyethylene) was inserted into the bladder for urine sample collection. After the surgical procedure, a continuous intravenous infusion of 0.9% NaCl and 3% mannitol at a constant rate of 100 μl/min was administered through an infusion pump (Harvard PHD 2000) for 30 min. After this period, a prime dose of inulin (Sigma) of 300 mg/kg body weight was administered, followed by a maintenance dose of 5 mg/min per kg body weight, through the infusion pump. The para-amino hippurate (PAH - Sigma) prime of 6.66 mg/kg of body weight was dissolved together with the prime dose of inulin, both being administered in the jugular vein. The maintenance dose of PAH was 1.33 mg/min per kg of body weight and was administered together with the maintenance dose of inulin. The glomerular filtration rate (GFR) and renal plasma flow (RPF) were evaluated based on the clearance of the respective substances (inulin and PAH). Blood samples were collected using heparinized syringes (Liquemine, Roche) and centrifuged at 5,000 rpm for 10 min (Fanem centrifuge, SP. B-204-NR), and plasma was separated and stored in a refrigerator until dosing. Urine samples were collected under mineral oil in previously weighed glass tubes. After collection, the tubes were weighed again to calculate urinary flow. Urinary flow values were normalized per kg. Afterward, the samples were refrigerated in collection tubes under mineral oil. Plasma and urinary concentrations of inulin and PAH were determined using a colorimetric method (Nascimento-Gomes et al., 1997).

The urinary and plasma concentrations of sodium (Na+) and potassium (K+) were determined using the flame photometry method (analyzer model 910).

Urinary osmolarity (Uosm) was evaluated using an osmometer (Advanced 3W2). Proteinuria was evaluated by the sulfosalicylic acid precipitation method (Nascimento-Gomes et al., 1997).

### Renal morphology

The kidneys were surgically removed under anesthesia with ketamine (65 mg/kg) and xylazine (6 mg/kg), fixed in Bouin’s solution (ethanol saturated with picric acid 75%, formaldehyde 20%, and acetic acid 5%), and embedded in paraffin. Five-micrometer histological sections were cut and stained with hematoxylin and eosin. Glomerular area was evaluated using a light microscope (Nikon H550L) and a camera. Images were analyzed using an image analysis software (Nikon, NISElements 3.2, Japan). Encircled areas were determined using computerized morphometry. Twenty fields were analyzed on each slide (magnification ×200). For immunohistochemical analysis, sections were incubated overnight at 4°C with the following antibodies: anti-CD68 for macrophage identification (anti ED1, 1:500, Serotec, Sigma-Aldrich, MO, United States); anti-alpha-SM-actin, 1:1000 (Dako, Glostrup-Denmark); anti-vimentin, 1:500 (Dako, Glostrup-Denmark); anti- endothelial nitric oxide synthase (eNOS), 1:250 (Gene-tex, CA, United States) and anti-8OHdG, 1:150 (Gene-tex, CA, United States); anti-sodium transporters: sodium-hydrogen exchanger 3 (NHE3), 1:300, sodium-potassium-chloride cotransporter 2 (NKCC2), 1:300, sodium-chloride cotransporter (NCC), 1:300, and epithelial sodium channel (ENaC), 1:300 (StressMarq Biosciences Inc. Victoria, BO, Canada); anti-renin, 1:75 (Santa Cruz Biotechnology, Dallas, TX, United States); anti-angiotensin converse enzyme 1, 1:400 and 2, 1:500 (Abcam Inc. Waltham - MA, United States). The reaction product was determined using a universal immuno-peroxidase polymer (Histofine-Nichirei Biosciences, Japan) or with Alexa Fluor 488, 1:500 (Invitrogen, Thermo Fisher Scientific, United States). For quantitative analysis, the percentage of the area was assessed in 20 consecutive fields for each sample (×200 magnification). Images were acquired using a microscope (Eclipse 80i, Nikon, Tokyo, Japan) equipped with a digital camera (DSRi1, Nikon) and analyzed using NIS-Elements (Nikon) software.

### Statistical analysis

Results are presented as mean ± standard error and were analyzed by one-way ANOVA. Additionally, Bonferroni’s post hoc test was used for multiple comparisons between groups (Prism 6.0, GraphPad). Values of *p* ≤ 0.05 were considered significant.

## Results

Table 1 presents the results obtained for the body weight, blood pressure, and renal function parameters of the experimental groups. There were no significant differences in body weights between the groups. Fructose intake had a positive effect on the blood pressure of male offspring of fructose dams that continued to receive fructose (group FF). The experimental group also presented a reduced GFR, sodium excretion, Uosm, increased proteinuria, and urine volume.

**TABLE 1:** Body weight, blood pressure and renal function parameters from the experimental groups.

Parameters	C (n=8)	FW (n=8)	FF (n=8)	ANOVA
Body weight (g)	407 ± 10.3	427 ± 12.8	446 ± 12.3	*p* = 0.0853
Kidney weight (g)	3.5 ± 0.12	3.9 ± 0.17	3.7 ± 0.11	*p* = 0.6309
Systolic blood pressure (mmHg)	121 ± 2.2	126 ± 1.3	137 ± 1.5*^#^	***p* < 0.0001**
Urinary volume (mL/24h)	12.1 ± 1.1	11.9 ± 0.6	39.3 ± 5.6*^#^	***p* = 0.0215**
Urine osmolarity (mOsm/L)	1617 ± 117	1108 ± 147*	348,2 ± 39*^#^	***p* = 0.0306**
Proteinuria (mg/24h)	5.5 ± 0.55	5.1 ± 0.22	9.7 ± 1.10*^#^	***p* = 0.0004**
Na^+^ excretion (µEq/min/kg)	1.12 ± 0.14	0.74 ± 0.12	0.40 ± 0.07*	***p* = 0.0010**
K^+^ excretion (µEq/min/kg)	0.99 ±0.08	4.10 ± 0.29*	1.72 ± 0.52^#^	***p* < 0.0001**
GFR (mL/min/kg)	7.8 ± 0.63	7.0 ± 0.66	4.9 ± 0.54*	***p* = 0.0085**
RPF (mL/min/kg)	16.0 ± 0.97	15.2 ± 1.26	15.8 ± 1.66	*p* = 0.2555

Differences statistically significant when *p* < 0.05 (bold); vs. control (C) * and vs. FW^#^ using ANOVA followed by Bonferroni. Values are means ± standard error, n = number of animals.

**FIGURE 1:**
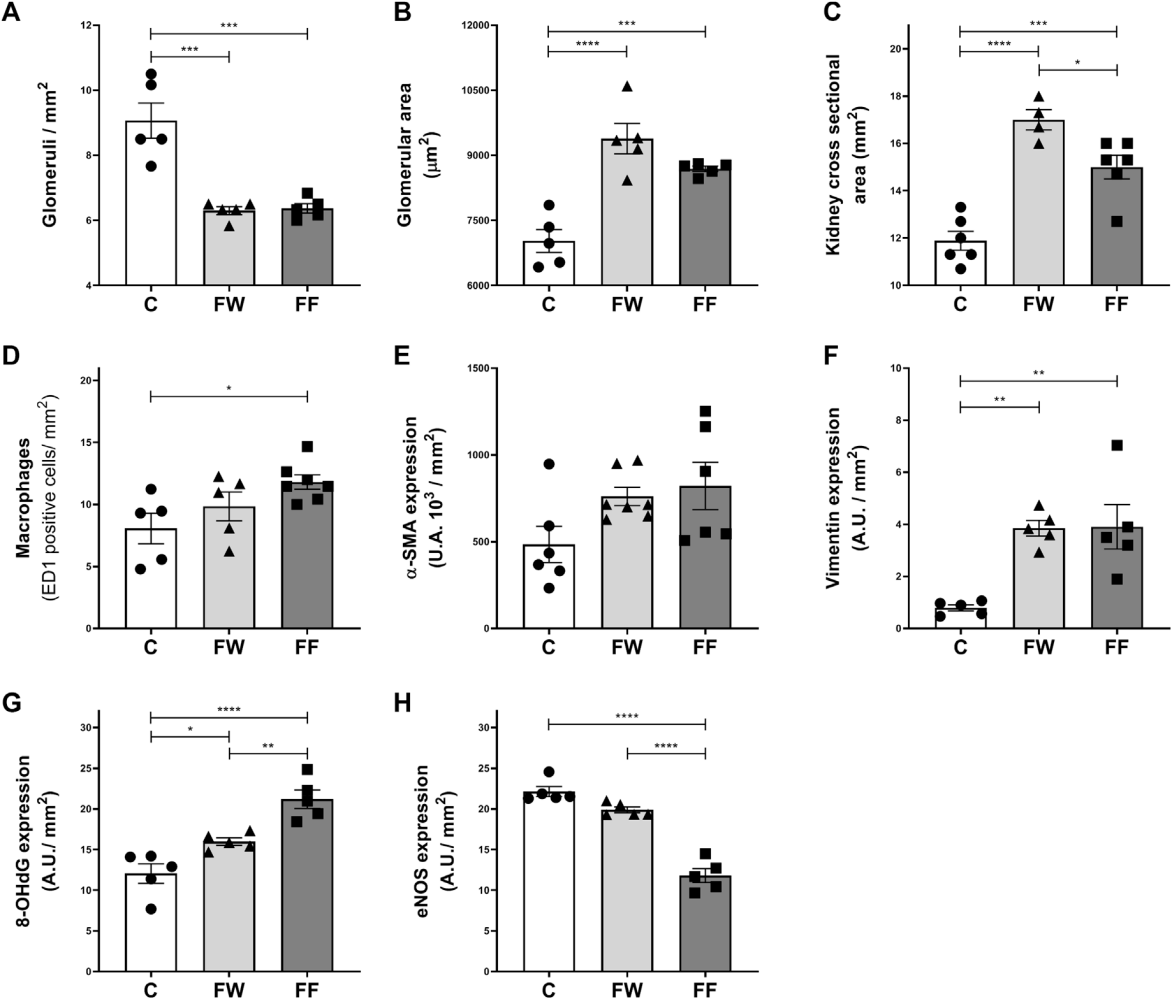
Kidney morphological parameters and expression of markers of renal dysfunction: Number of glomeruli/mm^2^ **(A)**; glomerular area **(B)**; kidney cross 398 sectional area **(C)**; macrophages **(D)** and expression of α-SMA **(E)**, vimentin **(F)**, 8-399 OHdG **(G)** and eNOS **(H)**. Significance level: ANOVA followed by Bonferroni; values are means ± standard error, 5–6 animals per group. * p ≤ 0.05; ** p ≤ 0.01; *** p ≤ 0.001; **** p ≤ 0.0001.

The morphological parameters of the kidneys are shown in Figure 1. Fructose intake during pregnancy caused a reduction in the number of nephrons (Figure 1A), glomerular enlargement (Figure 1B), and increased kidney cross-sectional area (Figure 1C). The treatment also increased macrophage infiltration (Figure 1D), vimentin (Figure 1F), and 8-OHdG expression (Figure 1G), but reduced the expression of eNOS (Figure 1H). Alpha-SM-actin and vimentin are proteins poorly expressed in the normal kidney. In renal injury these proteins are expressed by myofibroblasts after the epithelial-mesenchymal transition (EMT) (Sato and Yanagita, 2017). The expression of vimentin in EMT seems to play a modulator role in this process (Wang et al., 2018). Additionally, the production of pro-inflammatory cytokines and chemokines by myofibroblasts favors inflammation (Sato and Yanagita, 2017).

**FIGURE 2:**
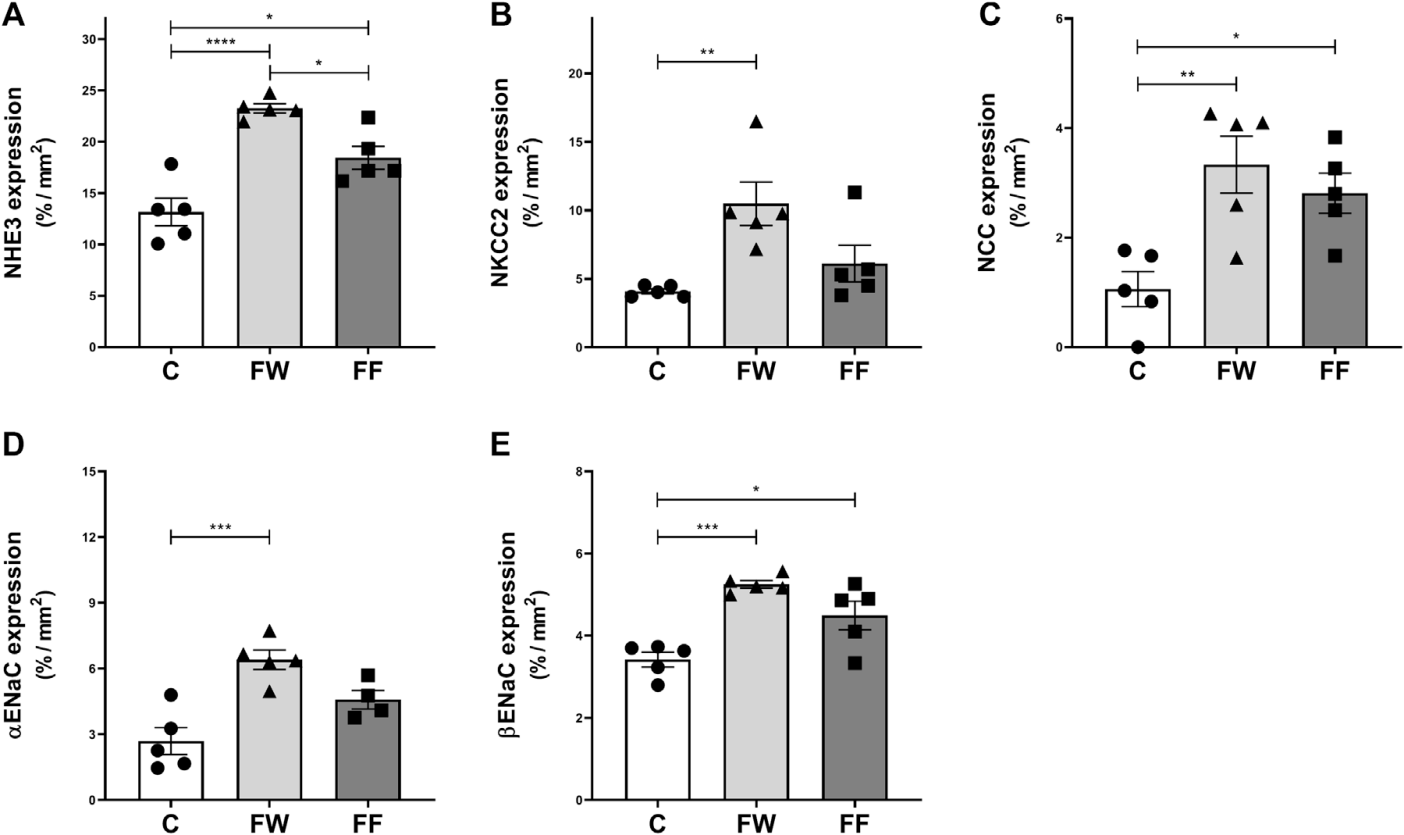
Expressions of renal sodium transporters: sodium-hydrogen exchanger 3 (NHE3) **(A)**, sodium-potassium-chloride cotransporter (NKCC2) **(B)**, sodium-chloride cotransporter (NCC) **(C)**, and epithelial sodium channel (α and β ENaC) **(D,E)**. Significance level: ANOVA followed by Bonferroni; values are means ± standard error, 5–6 animals per group. * p ≤ 0.05; ** p ≤ 0.01; ***p ≤ 0.001; **** p ≤ 0.0001.

**FIGURE 3:**
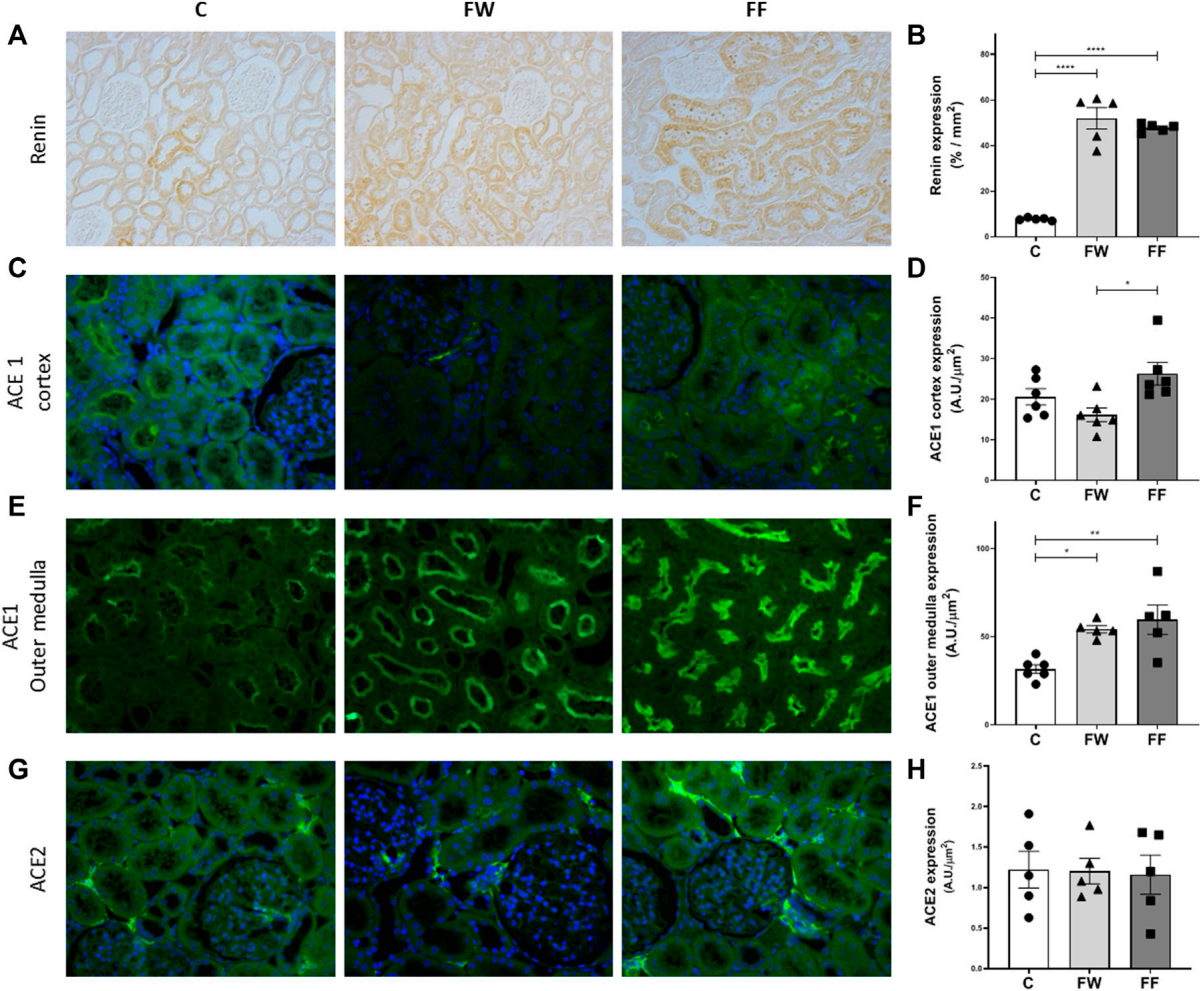
Renal expressions of renin, ACE1 and ACE2. Representative photomicrographs and quantitative analysis of the renin **(A–B)** (original magnification ×200; by immunohistochemistry); ACE1 expression in the cortex **(C–D)** and outer medulla **(E–F)**; and ACE2 **(G–H)** (original magnification ×400; by immunofluorescence). Signficance level: ANOVA followed by Bonferroni; values are means ± standard error, 5–6 animals per group. * p ≤ 0.05; ** p ≤ 0.01; *** p ≤ 0.001; **** p ≤ 0.0001.

The expression of renal sodium transporters is shown in Figure 2. Increased expression of all evaluated transporters was observed in the FW group. The FF group showed an increased expression of NHE3, NCC, and βENaC. The expression levels of the renin-angiotensin-system (RAS) components: renin, angiotensin-converting enzymes (ACE) 1, and ACE2, are shown in Figure 3. The expression levels of renin (Figure 3B) and ACE1 (Figure 3F) significantly increased in the FW and FF groups. Figures 3A,C–E show the representative illustrations of the histological sections.

## Discussion

The impact of high fructose consumption during pregnancy and lactation on blood pressure, renal morphology, and function in offspring was evaluated in the present study. The most significant changes were observed in the FF group (offspring of fructose dams that continued to receive fructose after weaning). These animals presented with elevated BP and functional alterations, such as a reduction in glomerular filtration rate, urinary osmolarity, and increased urinary protein excretion. The morphological alterations found in FF were glomerular hypertrophy, macrophage infiltration, increased expression of renal sodium transporters (NHE3, NCC, and ENaC), and increased expression of renin, ACE1, and the DNA stress marker (8-OHdG). The FW group (offspring from fructose dams that received water to drink after weaning) did not show increased BP or renal functional alterations; however, they presented glomerular hypertrophy, macrophage infiltration, increased expression of renal sodium transporters (NHE3, NKCC2, NCC, and ENaC), and increased expression of renin and ACE1. These changes are a consequence of an altered intrauterine environment and may have repercussions in the future.

The elevation of BP resulting from the consumption of fructose remains the focus of many studies. Chronic activation of the sympathetic nervous system (SNS) is one of the mechanisms responsible for this effect. In fructose-fed mice with a chronic record of BP by radiotelemetry, spectral analysis of the heart rate and pressure showed that sympathetic modulation to the heart and vessels was increased, confirming autonomic imbalance (De Angelis et al., 2012). In another study, the use of the α1-adrenergic receptor antagonist prazosin in fructose-fed rats prevented the development of hypertension and normalized norepinephrine levels, confirming the participation of SNS in the increase in BP in this experimental model (Tran et al., 2014). Furthermore, in rats treated with fructose, along with the elevation of BP, there was a significant increase in the sympathetic activity of the renal nerve (SNRA), and renal denervation of these rats resulted in the normalization of BP values (Soncrant et al., 2018). The factors triggering sympathetic activation by fructose are not yet well defined. It is speculated that this sugar is involved in the central nervous system and the effects of increased insulinemia (Klein and Kiat, 2015; Spagnuolo et al., 2020).

Moreover, it is known that the increased sympathetic activity stimulates the renal release of renin and activates the RAS, which also favors the elevation of BP (DiBona, 2000; DiBona, 2001; Sata et al., 2018). Fructose overload seems to alter the expression of RAS components and increase the production of angiotensin II in the kidneys (Yokota et al., 2018) and heart (Froogh et al., 2020). In addition to the effects on BP, an imbalance in RAS during fetal development can alter kidney formation, resulting in reduced nephron number (Yosypiv, 2021). In the present study, maternal fructose overload caused reduced nephron numbers in both the FW and FF groups. This alteration may be related to changes in RAS during pregnancy. However, maternal fructose can also alter fetal development by reducing placental growth (Asghar et al., 2016) and increasing fetal oxidative stress because fructose crosses the placental barrier and reaches the fetal blood circulation (Liu et al., 2021). However, further experiments are necessary to confirm the role of these mechanisms in the present experimental model.

In addition to the reduction in nephron number, the FW and FF groups presented glomerular hypertrophy. According to Brenner et al. (Brenner et al., 1988; Brenner et al., 1996; Brenner and Mackenzie, 1997), in individuals with fewer nephrons, glomerular hypertrophy is a compensatory mechanism to increase glomerular ultrafiltration. This increase in glomerular filtration can be classified as “relative glomerular hyperfiltration” because it occurs in a reduced number of nephrons. In both conditions: “absolute” and “relative” glomerular hyperfiltration, there may be structural damage to the glomeruli. The increase in glomerular capillary hydraulic pressure, causes stretch in the capillary wall, and creates tensile stress and shear stress, leading to changes in the glomerular basement membrane that aggravate over time. Other factors also contribute to glomerular damage (Cortinovis et al., 2022). Together with glomerular enlargement, renal hypertrophy was observed in both the FF and FW groups, as evidenced by the increase in the renal cross-sectional area. In the FW group, we could infer that glomerular hypertrophy contributed to maintaining GFR close to the control group values. However, in FF, fructose intake caused further changes, resulting in a reduced GFR. Additionally, the FF group showed increased proteinuria, which is a sign of glomerular damage (Singh et al., 2009). The experimental group also exhibited increased urinary flow and reduced Uosm. These changes are indeed related to higher water intake due to the sweet taste of fructose. The FW group also had lower Uosm than the C group, suggesting a deficit in the mechanism of urinary concentration. Using a fetal programming model, Madsen et al. (2010) demonstrated that changes in the RAAS during nephrogenesis modify the vascular architecture of the medulla, impairing the urinary concentration mechanism (Madsen et al., 2010). We intend to investigate the alterations in medullar morphology in the present experimental model.

Another functional alteration observed in FF is a reduction in sodium excretion. Sodium retention has been identified as a factor leading to arterial hypertension in animals treated with fructose (Soleimani and Alborzi, 2011). To evaluate the role of renal sodium transporters in the observed results, the expression of these proteins was assessed (Figure 2). Increased expression of NHE3, NCC, and ENaC was observed in the FF group, confirming their participation in sodium retention induced by high fructose intake. Nonetheless, in this group (FF), NKCC2 expression did not increase. In these animals, the higher water intake must have reduced vasopressin secretion, which, under normal conditions, exerts a stimulatory effect on NKCC2 (Xue and Tang, 2016); thus, in the absence of this stimulus, NKCC2 expression was not augmented. However, this group received fructose until the end of the experimental period; therefore, the alterations of the transporters may be due to the direct effect of fructose in addition to the effect of maternal fructose intake. Similar results were observed in rats treated with fructose by Xu et al. (2017) and Ares et al. (2019) and may be linked to changes in the renal expression of RAS components (Xu et al., 2017; Ares et al., 2019; Xu et al., 2021). In the FW group, the expression of all sodium transporters, including NKCC2, increased. This confirms that the fetal environment, under the influence of fructose, modifies kidney development and function, possibly due to alterations in RAS. To further understand these effects, Seong et al. (2019) evaluated the serum concentration and expression of RAS components in the renal tissue of the offspring of mice treated with fructose during pregnancy and lactation. These authors observed increased serum concentrations of renin, angiotensin II, and aldosterone, higher blood pressure values in the offspring (first generation), and increased mRNA expression of the angiotensin II type 1b receptor (AT1b) and NCC sodium transporter. In a similar experimental protocol, Koo et al. (2021) confirmed the increase in blood pressure in the offspring; however, they did not observe augmented serum concentrations of renin, angiotensin II, and aldosterone, nor altered mRNA expression of RAS components or sodium transporters in these mice (Koo et al., 2021). Thus, the effects of maternal fructose intake during pregnancy on the expression of RAS components in the kidneys of offspring are still not defined.

To evaluate the effect of maternal fructose intake during pregnancy on the expression of RAS components in the kidneys of the offspring, we evaluated the expression of ACE1, ACE2, and renin. Increased renin expression was observed in both FF and FW; this expression was located mainly in tubular cells. In addition to its synthesis and release by juxtaglomerular cells, renin is also produced by the principal cells of the connecting tubules and collecting ducts in rat, mouse, and human kidneys (Hackenthal et al., 1990; Rohrwasser et al., 1999; Prieto-Carrasquero et al., 2004). In experimental models with high levels of angiotensin II (ANG II), juxtaglomerular renin is suppressed, although its expression increases in the distal nephron (Gonzalez et al., 2017). Renin produced in the late nephron segments may act on angiotensinogen delivered from proximal segments, generating angiotensin I, which, under the action of ACE1 existing in the collecting duct, may contribute to the increase in the generation of ANG II (Casarini et al., 1997; Casarini et al., 2001). In hypertensive rats (Goldblatt model), increased ACE1 expression, in association with elevated renin activity in the late segments of the nephron, resulted in augmented ANG II content, suggesting an additional mechanism for the increase in intrarenal ANG II in this experimental model (Prieto et al., 2011). We also observed an increased expression of ACE1, mainly in the outer medulla, in the FW and FF groups. This increase could have contributed to the increase in intrarenal ANG II production in this experimental model; however, additional experiments are needed to confirm this hypothesis. Thus, exposure to fructose during fetal development appears to positively modulate renin and ACE1 expression, possibly related to the increased production of intrarenal ANG II, contributing to the observed renal changes in this experimental model.

Other changes observed in the kidneys of FW and FF rats included increased macrophage infiltration, increased expression of the oxidative stress marker 8OHdG, and reduced expression of the endothelial nitric oxide (NO) synthase enzyme. Dietary fructose is absorbed by enterocytes and reaches the blood stream. In the liver, it is initially phosphorylated by ketohexokinase (KHK), and then undergoes the action of several enzymes, giving rise to uric acid (Choi et al., 2010). In renal epithelial cells, fructose triggers proinflammatory pathways by inducing MCP-1 production, mediated by KHK, and MCP-1 production can also be induced by uric acid and reactive oxygen species (ROS). ROS production can come from two main sources: NADPH oxidase and xanthine oxidoreductase (Cirillo et al., 2009). Fructose-induced uric acid production can also cause uncoupling of eNOS and reduce the availability of NO (Nakagawa and Kang, 2021). Our results confirmed that high fructose intake increased macrophage migration to the kidneys, augmented ROS production, and decreased NO synthase enzyme expression, which may be associated with uric acid production but may also be related to the upregulation of RAS components. Further experiments are necessary to confirm this hypothesis.

## Conclusion

Fructose overload during pregnancy impacted the fetal environment, causing kidney morphological changes in the offspring, such as reduction in the number of nephrons and glomerular hypertrophy observed in adulthood. Maintaining fructose overload accentuated the effect on renal function, raising blood pressure, reducing glomerular filtration rate and increasing proteinuria. The enhanced expression of RAS components in the final segments of the nephron possibly contributed to the renal alterations observed in this experimental model.

## Data Availability

The original contributions presented in the study are included in the article/supplementary materials, further inquiries can be directed to the corresponding author.
