# Supplementary material for: Effects of maternal fructose intake on the offspring’s kidneys
Source: Front Physiol. 2022 Sep 6;13:969048. doi: 10.3389/fphys.2022.969048 (PMC9485812; doi:10.3389/fphys.2022.969048)

Supplementary data - Figure 1. Kidney morphological parameters: representative photomicrographs of kidney (**A**) from control offspring (C), fructose offspring that received water to drink (FW), and fructose offspring that received fructose solution to drink (FF) (original magnification 200×; HE staining); and kidney cross sectional área representative images (**B**).

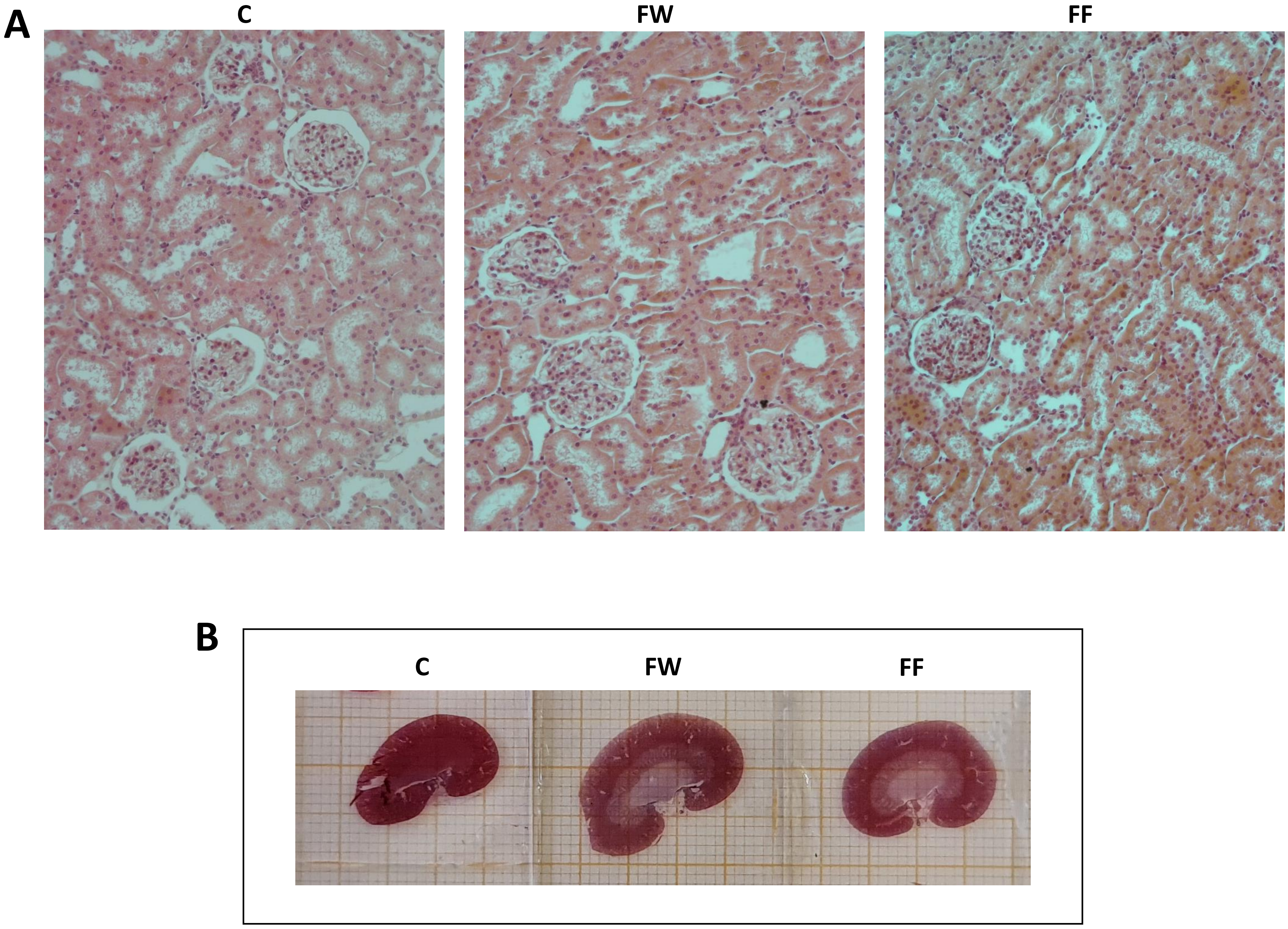

Kidney cross sectional area

Supplementary data - Figure 2. Expression of markers of renal dysfunction: representative photomicrographs and quantitative analysis by immunohistochemistry of the macrophages (**A-B**) (ED1 positive cells – arrows; original magnification 400×); alpha-SM-actin (α-SMA) (**C-D**), vimentin (**E-F**), 8-OHdG (**G-H**); and eNOS (**I-J**); (original magnification 200×). Significance level: ANOVA followed by Bonferroni; values are means ± standard error, 5-6 animals per group* $p < 0.05$; ** $p < 0.01$; *** $p < 0.001$; **** $p < 0.0001$.

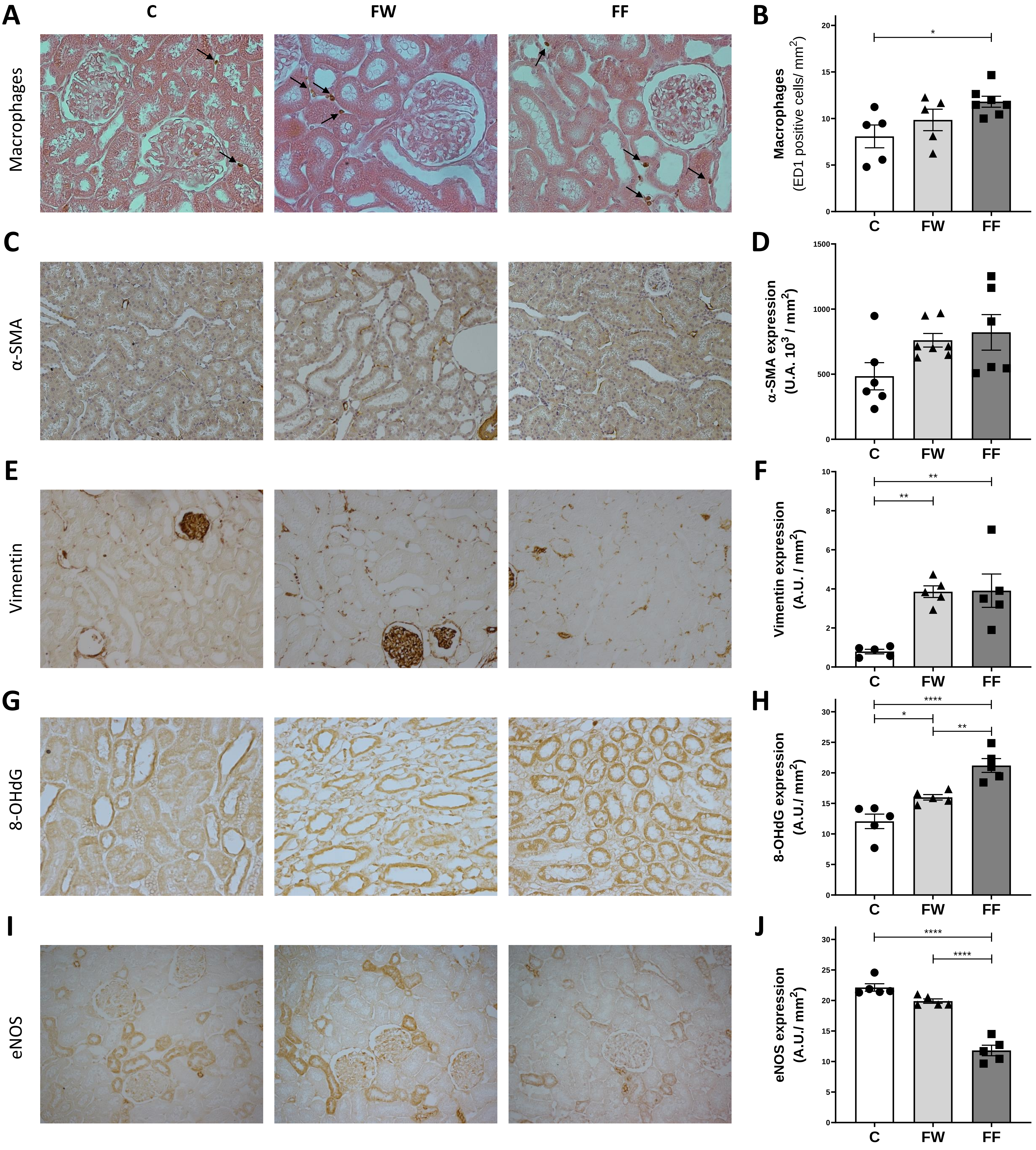

Supplementary data - Figure 3. Expressions of renal sodium transporters: representative photomicrographs and quantitative analysis (original magnification 200×; by immunohistochemistry) of the sodium-hydrogen exchanger 3 (NHE3) (**A-B**); sodium-potassium-chloride cotransporter 2 (NKCC2) (**C-D**), sodium-chloride cotransporter (NCC) (**E-F**), and epithelial sodium channel (α and β ENaC) (**G-H** and **I-J**). Significance level: ANOVA followed by Bonferroni; values are means ± standard error, 5-6 animals per group* p < 0.05; ** p < 0.01; *** p < 0.001; **** p < 0.0001.

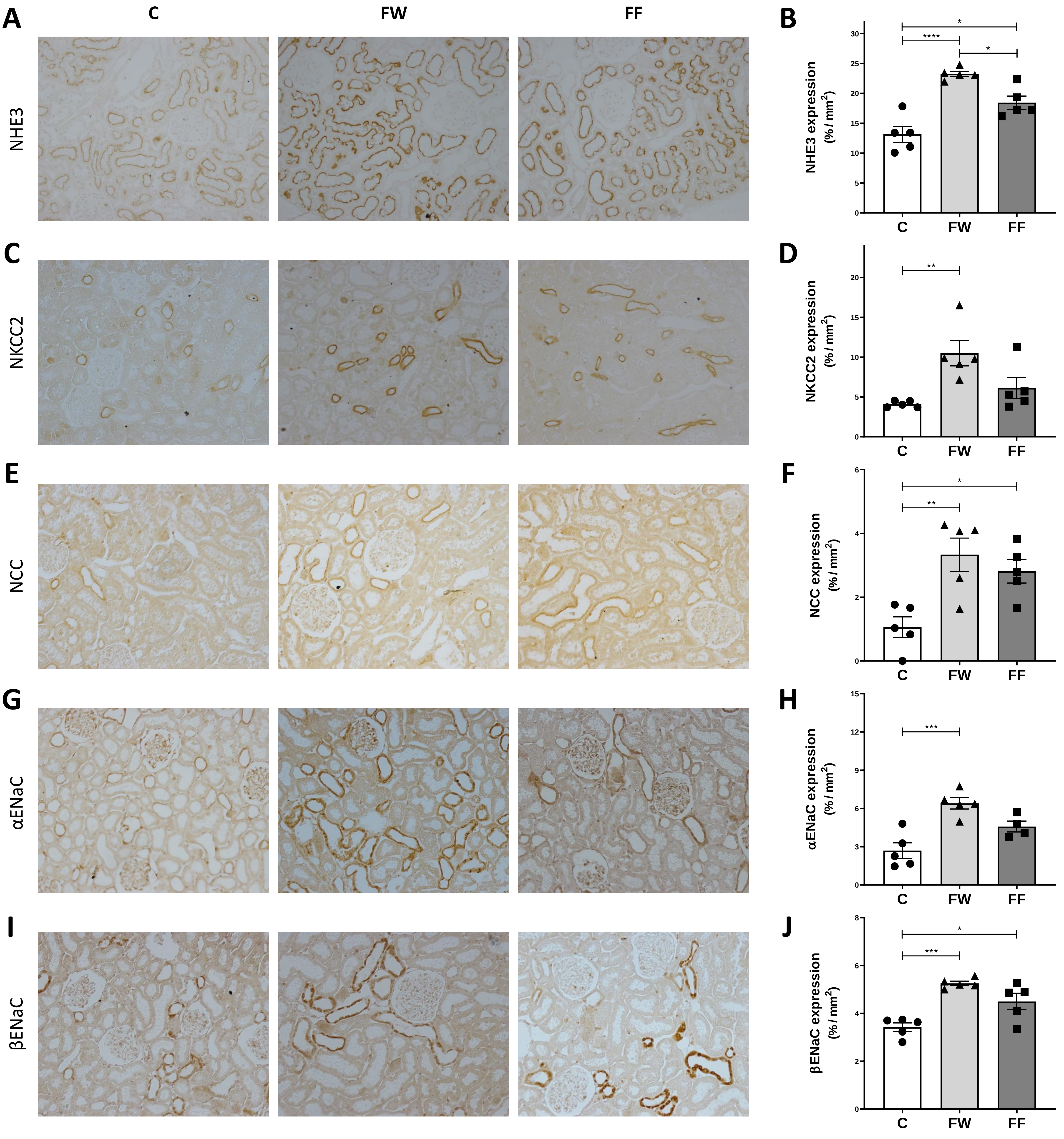

Supplement: Supplementary file 1 [file DataSheet1.PDF]
